# Enhancing Mergers and Acquisitions (M&A) performance: Analyzing the role of human resource practices in Sri Lanka’s telecommunication industry through Lewin’s change management model

**DOI:** 10.1371/journal.pone.0317117

**Published:** 2025-01-10

**Authors:** S. H. N. Pubodhya, Wasantha Rajapakshe

**Affiliations:** SLIIT Business School, Sri Lanka Institute of Information Technology, Malabe, Sri Lanka; National University of Sciences and Technology, PAKISTAN

## Abstract

In recent years, the Sri Lankan telecom industry has experienced significant expansion and transformation, leading to a notable increase in mergers and acquisitions (M&A). This study investigates the impact of human resource practices on M&A performance, utilizing Lewin’s Change Management Model as a framework. The research is based on a questionnaire survey, examining factors influencing the performance of M&A in Sri Lanka Telecom industry, such as communication (Unfreeze), training (Change), leadership (Refreeze), and performance (M&A outcomes). Structural Equation Modeling (SEM) reveals that communication significantly influences training (β = 0.800), while training has a strong effect on leadership (β = 1.062), both directly and indirectly via communication (β = 0.850). Additionally, performance is positively impacted by training (β = 0.819) and leadership (β = 0.459), with communication exerting a substantial indirect influence on performance (β = 0.655). These results underscore the necessity for an integrated approach that enhances organizational performance and adaptability in an evolving business landscape. To optimize M&A outcomes, organizations are encouraged to prioritize leadership development, invest in comprehensive training programs, and align communication strategies effectively. This research contributes valuable insights into the management of change within the telecom sector, promoting sustainable growth and success in future M&A endeavours.

## Introduction

The telecommunication industry in Sri Lanka has significantly transformed in recent years, with mergers and acquisitions (M&A) emerging as a transformative strategy. This sector, characterized by dynamic growth, relentless technological innovations, and increasing customer demands, has catalysed for societal progress and economic development. M&A has played a pivotal role in this transformation, enabling telecommunication companies to not only expand their market share and bolster their competitive positions but also to meet the increasing customer demands, thereby reassuring the industry’s responsiveness to market needs [1].

M&A activities, with their potential to significantly reshape industry landscapes, also present complex challenges. The successful execution of M&A relies on many factors, with human resource practice occupying a central role. Recognizing the vital role of human resource practices is essential, as these practices are crucial in influencing employee engagement, organizational culture, and strategic alignment [2].

There are many failure stories related to M&A around the globe. America Online Inc. announced its plans to buy Time Warner Inc. These two companies had cultural challenges. The dot-com bubble burst shortly after the deal, causing the company’s AOL segment to lose much value [3]. Sprint Communications acquired a majority stake in Nextel Communications to become the third-largest telecommunications provider in the United States of America. These two companies had technological differences and had zero overlaps, meaning they had no common areas or functions, making integration a failure. They also found it difficult to merge operations and had clashing marketing strategies, eventually allowing rival companies to steal dissatisfied customers. Nextel employees often had to seek approval from Sprint’s higher-ups to implement corrective actions [4]. The lack of trust and rapport meant many such measures needed to be approved or executed correctly. Customer service suffered greatly, and Sprint struggled to keep its customers happy. As a result, the acquisition failed, and they lost a large amount of market share. However, many Asian countries liberalized their policies and introduced many strategies, including M&A, to enhance the efficiency of the service [5].

The recognition of M&A success stories within the Sri Lankan telecommunication context underpins the motivation to embark on this research journey. In the recent past, Mobitel Pvt. Ltd, a leading network provider in Sri Lanka, has merged with Sri Lanka Telecom PLC, a leading telecommunication provider in Sri Lanka. This successful merger not only serves as an inspiration but also as a driving force, igniting the potential benefits of M&A activities in the Sri Lankan telecommunication industry [6].

The acquisition of e-Channeling E-Channeling (Pvt) Ltd by SLT Mobitel in 2016 was a significant milestone. SLT Mobitel, Sri Lanka’s second-largest mobile telecommunications operator, required 87.6% of E-Channeling PLC from Senior Marketing System Asia PTE for LKR 732Mn in October 2016, as per corporate statements. This strategic move not only expanded Mobitel’s reach but also promised a host of new products and services for all mobile customers, not just health and medical, thereby enhancing the overall customer experience [7, 8].

In December 2018, CKHG subsidiary Hutchison Telecommunications Lanka (Hutch Lanka) acquired and consolidated Etisalat Lanka from Emirates Telecommunications Group Company (Etisalat Group). This acquisition will result in CKHG owning 85% of Hutch Lanka and Etisalat Group 15%. The most significant outcome of this acquisition is the improved coverage that Hutch and Etisalat subscribers will enjo**y** from the new network [9]. This is a positive development that all stakeholders can support.

Dialogue Axiata acquires the leading Microsoft solutions provider H One Ltd, opening up new growth opportunities. Axiata’s wholly owned subsidiary, Dialogue Broadband Network (Private) Limited (DBN), bought 100% of H One (Pvt) Ltd in January 2021. This acquisition makes commercial and strategic sense for Dialogue, Sri Lanka’s leading telecoms carrier with a dominant corporate and enterprise market share. In pandemic-driven economies where corporations large and small are rapidly digitizing their activities, this M&A will undoubtedly benefit Dialogue [7].

The empirical evidence demonstrates that most mergers and acquisitions in the telecommunications business in Sri Lanka have attained the intended results [7, 10]. Therefore, assessing and determining the factors that impact success is crucial. The decision to undertake this study has been informed by the pressing need to gain deeper insight into the interplay between human resource practices and M&A performance in this context. Moreover, it is motivated by the desire to offer a comprehensive understanding of how telco companies can effectively manage their human resources during M&A, mitigate challenges, and leverage opportunities for enhanced performance paving the way for a more successful telecommunications industry.

Inadequate human resource practices have resulted in cultural clashes and integration issues, talent drain and retention problems, employee resistance and low engagement, loss of focus on core business operations, and delayed synergy achievement during M&A activities in the telecommunication industry in Sri Lanka [11]. This research is highly relevant as it aims to study the extent to which human resource practices impact M&A performance in the telecommunication industry in Sri Lanka, a topic of significant interest and importance.

### Contribution of the study

This study has significant implications and contributions to the telecommunications industry, where M&A is becoming an increasingly prevalent strategic growth option. The practical insights offered by this research are directly applicable to industry practitioners. By understanding the impact of human resource practices on the performance of M&A activities, telecommunication companies can make informed decisions, mitigate risks, and optimize the integration of their human resources, thereby enhancing overall performance. The findings of this study serve as a practical guide for industry leaders seeking to navigate the intricate terrain of M & A in the telecommunication sector.

Effective human resource practices are the core foundation of a successful organization. By focusing on specific HR practices such as cultural integration strategies, leadership development programs, and employee retention initiatives, this research can provide telecommunication companies with strategies for improving their leadership transitions, talent management, and employee engagement during transitions. This can lead to smoother integration, minimized disruptions, and improved overall operational performance.

This study offers valuable insights for policymakers, highlighting their crucial role in informing the development of policies and guidelines that will promote best practices in HR management during M&A. Such policies, guided by their influence, can help ensure that the broader societal and economic benefits of a healthy telecommunication sector are safeguarded during industry transactions.

This research significantly contributes to understanding the relationship between human resource practices and M&A performance in the context of M&A. The practical implications of the findings can be directly applied by telecommunication companies and industries undergoing similar transformations. This research, therefore, equips the reader with actionable insights and serves as a valuable reference point for multisector organizations throughout the globe engaged in M&A activities.

### Theoretical perspectives

**Fig 1:**
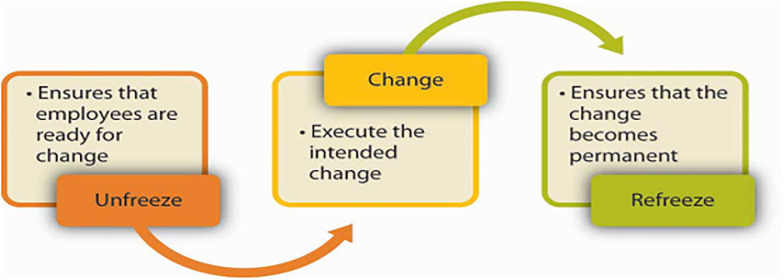
Lewin’s change model. Source: [12].

Kurt Lewin’s Change Management Model (1940) (Unfreeze-Change-Refreeze) is employed to support the theoretical framework of this research, a model that has stood the test of time and research and will be expounded accordingly [12]. This model highlights the importance of preparing employees for change (unfreezing), implementing change effectively, and reinforcing new behaviors (refreezing) (Fig 1). Furthermore, researchers have periodically incorporated additional elements into the model based on their research, further enhancing its credibility [13]. This study utilizes Lewin’s Change Management Model to identify how HRM practices influence organizational change during M&A [14]. The organization’s approach to managing the transition may differ based on the level of preparedness and willingness to embrace the change. Consequently, the change process in the telecommunication industry is explained in three stages, as proposed by the Lewin model (Fig 1): unfreezing, altering, and refreezing.

The unfreezing phase, a crucial stage in the change process, involves preparing employees for change by unfreezing their current mindset and organizational practices. This stage is where HR professionals play a pivotal role. Initially, individuals tend to instinctively resist change. Employees need to understand alterations. Organizations are unable to effectively communicate changes to employees because of abrupt government actions. There are deliberate and unexpected alterations. Certain modifications were unforeseen, and the crew lacked sufficient expertise. Employees were perplexed and inundated with unforeseen occurrences. HRM practices that can be included in this level are to change communication strategies [14]. HR should communicate clearly and consistently about the reasons for the M&A, its expected impact on the organization and employees, and the timeline for implementation. This helps employees understand the need for change and reduces uncertainty. Equally important is involving employees early in the process, making them feel included and part of the change journey. Their involvement in decision-making and planning processes related to the M&A can enhance acceptance and readiness for change.

The changing phase, a critical stage in the change process, focuses on implementing the changes required by the M&A, such as integrating systems, processes, and cultures. Most individuals find it challenging to cope with the actuality of their circumstances. They are providing comprehensive training programs that equip employees with the new skills, knowledge, and competencies needed in the post-M&A environment. Communication breakdowns, new policies and procedures, technology, and new management tormented employees. This ensures employees are prepared to perform effectively in their revised roles and responsibilities. Significantly, establishing dedicated teams or change agents within HR and across departments plays a crucial role in facilitating the implementation process, addressing challenges, and providing ongoing support to employees, making them feel reassured and guided.

In the Refreezing phase, the changes introduced during the M&A are institutionalized and reinforced to become the new normal. This phase is where continuous communication becomes crucial. Work must align with the prevailing organizational culture and meet the standards of acceptability. The group asserts that rewarding acts can lead to their repetition through positive reinforcement and individual effort. HRM practices can include reinforcing new behaviors through inspiring leadership and continuous communication. Maintaining open lines of communication to reinforce the rationale behind the changes and to address any lingering concerns or resistance. HR can conduct regular assessments and feedback sessions to evaluate the effectiveness of the changes, gather insights for further improvement, and ensure that refreezing is sustained over time.

Mergers and acquisitions compel several organizations, including corporations, to embrace a new corporate culture. This shift is not just a change in structure, but a transformation that affects the very essence of the organization. The reorganization of the M&A department resulted in staff experiencing uncertainty and stress. Insufficient guidelines, protocols, and communication during training and guidance can lead to demotivation, stress, and job insecurity. Employees’ effectiveness during organizational transition in M&A depends on leadership, communication, training, motivation, and change tolerance [15]. The HRM practices linked to Lewin’s Change Management Model enhance employee readiness, involvement, and acceptance of merger and acquisition changes, enhancing integration effectiveness, thereby underlining the urgency and importance of the issue.

### Literature review

In telecommunications, mergers and acquisitions (M&A) enhance competitiveness, achieve scale, and expand market share. However, it is human resource (HR) management that plays a pivotal role in the success of these transactions. Recent attention in Sri Lanka’s telecommunications industry has focused on how HR standards influence M&A performance. Research indicates that effective HR practices, such as communication and training, significantly shape the outcomes of M&A activities.

#### Communication.

The scenario of the success of M & A in the communication industry can be depicted through Kurt Lewin’s change model [12]. In the first stage, many people naturally resist change. The changes must be communicated well to employees. However, the role of planned changes is crucial in maintaining employee awareness and providing a sense of control. There are both unplanned and planned changes made. Some of the changes were unplanned, and employees’ awareness of the changes was not up to that level. Employees were confused by sudden changes and could not adapt to them. Thus, in the **unfreezing phase, communication** is essential.

Communication, particularly its various aspects such as openness, effectiveness, scope, continuity, richness, and approaches, plays a crucial role in the success of mergers and acquisitions (M&A) [16]. This study investigates their impact on M&A outcomes, particularly employee attitudes and the survival of the venture. Analyzing the Nigerian banking sector shows that effective communication, notably in reducing uncertainty, facilitates transitions, and boosts post-M&A commitment and success rates. The research fills gaps in HR and M&A literature by highlighting the importance of different communication types and examining the complex relationship between communication tactics and M&A outcomes.

Communication is not just important, but vital for the success of Chinese cross-border mergers and acquisitions (CBMA) post-M&A success [17]. This study by exploring factors like communication expediters, language barriers, acquisition experience, trust, business relatedness, and investor types in Chinese CBMAs, underscores the critical role of communication. It showcases best practices, demonstrating that communication accuracy, efficiency, and international management are key to enhancing organizational commitment and integration success across national contexts.

Another study investigates how M&A communication modalities impact employee emotions. It reveals that management communication and information dissemination during M&A trigger events that influence both positive and negative emotions. These emotions, in turn, affect employee attitudes, behaviors, and performance, ultimately impacting M&A success. Recognizing this relationship is crucial as it enlightens companies about the impact of M&A strategies on employee satisfaction [18].

Communication and managerial support significantly influence employee engagement and success during M&As. In the Philippines, poor communication exacerbates cultural differences, uncertainties, and conflicts, leading to higher employee turnover and negatively affecting M&A performance. This study underscores the importance of effective communication in cross-cultural management during M&As [19].

The Indonesian oil and gas industry is investigated to determine the factors **that influence** post-M&A performance. This study places a significant emphasis on the role of communication and compensation. M&A emerged as a vital strategy for market positioning and profitability, with recent agreements and values fluctuating to and enhance competitiveness in Indonesia. Employee performance is a crucial factor in M&A success, and effective communication before and after M&A is a key element for employee integration. The study supports the connection between communication, remuneration benefits, and employee performance, revealing factors that enhance M&A performance [20].

Analyzing the impact of M&A on shareholder value across 100 publicly listed Swedish companies with global operations involves examining several vital aspects. The study underscores how communication significantly influences M&A performance, examining factors such as effective communication, tonality, strategic alignment, and language’s positive and negative aspects in organizational decision-making. These profound findings not only highlight communication’s critical role in the dynamic global M&A landscape but also offer valuable stakeholder insights [21], enlightening the reader on the complex dynamics of M&A.

Investigating the impact of external business model communication on the financial performance of ten Danish cross-national M&As has uncovered significant deficiencies in M&A process business model communication and a weak correlation between above-average storytelling communication and corporate performance. These findings underscore the crucial and urgent need for skilled and deliberate communication strategies in M&As to safeguard the interests of all parties involved [22].

#### Training.

Recent literature extensively discusses HR practices, particularly training, in enhancing M&A performance. According to Lewin’s model, the second stage involves unfreezing people to transition into new stages. Effective training, communication, support, and time help employees adapt to new policies, technologies, and management, which are crucial for successful M&A integration. This reiterates the importance of effective training in M&A performance, convincing the audience of its necessity.

Training is crucial in Lewin’s Change Model [12, 23]. During the unfreezing stage, training creates awareness and readiness by explaining the rationale behind the change and addressing concerns, reducing resistance [24]. However, it is hands-on training, simulations, and continuous support that truly build competence, instilling a sense of confidence in the audience about the effectiveness of these training methods.

Effective communication plays a crucial role in training, as it ensures that the trainees clearly understand the objectives, procedures, and expectations, leading to better outcomes. The benefits of clear communication in training programs are immense, as it enhances the comprehension and retention of training material. This study found that when instructors communicated effectively, using clear, concise language, and providing opportunities for questions and feedback, trainees were more likely to successfully understand and apply the training content [25].

Organizations with solid communication practices tend to have more effective training programs. The report illustrated that effective communication helps reduce misunderstandings, align training with organizational goals, and ensure that trainees feel supported and engaged throughout the learning process [26].

These examples underscore the significant impact of effective communication on the success of training programs. Effective communication helps deliver content more effectively, address trainees’ concerns, and enhance performance. Moreover, many scholars have identified the importance of training in M&A performance.

Employee training emerges as a critical component for successful M&A integration. Training interventions are not just a formality, but a crucial step that facilitates cultural alignment, knowledge transfer, and employee adaptation, thereby enhancing integration outcomes [27]. Training in the changing phase is not just about initial adaptation but also about ongoing learning and improvement. Organizations can use feedback from training sessions to refine processes, address emerging challenges, and capitalize on opportunities for innovation and efficiency. This underscores the critical yet often overlooked relationship between HR practices and M&A outcomes during integration phases [28]. It emphasizes the significance of training in Israel’s context, where a high proportion of scientists and engineers underscores the importance of human capital in economic growth and technological advancement.

Effective training not only aligns individual and team efforts with the strategic objectives of M&A, but also underscores the crucial role of employees in the integrated organization. This understanding of their significance enhances productivity, efficiency, and commitment during the changing phase. While financial and strategic assessments are common in M&A due diligence, understanding the target organization’s culture, including training programs, is often neglected. They propose a comprehensive framework that integrates training with cultural elements to identify integration challenges and synergies, which is crucial for addressing the high failure rates observed in M&A activities [29].

Training programs in the changing phase also focus on changing management principles and practices. Employees are prepared to cope with uncertainty, manage resistance to change, and adopt new working methods. This builds organizational resilience and reduces the disruptions that can occur during the integration process. The impact of training on HR strategies during M&A integration. Their research emphasizes the role of leadership, change management, and HR retention strategies in optimizing training practices to enhance integration outcomes. They underscore the importance of training plans and cross-training in leveraging shared HR resources across borders [30].

Providing training during the changing phase is a clear demonstration of organizational investment in employees’ development, boosting engagement and morale and reducing turnover. The complexities of cross-border M&A, such as cultural integration and emotional resilience, underscore the importance of training in equipping employees, including senior management, with the necessary skills and competencies to navigate organizational change successfully [31].

Mergers and acquisitions often involve the challenging task of combining different organizational cultures. Training plays a crucial role in fostering cultural integration by educating employees about the newly integrated organization’s values, norms, and behavioral expectations. This not only promotes understanding and cooperation among employees from different backgrounds but also significantly enhances collaboration during the transition. The study in the Nepalese banking sector highlights how M&A affects employee performance. This study emphasizes the importance of training, work culture, and performance appraisals in facilitating employee adaptation and organizational success post-M&A [32]. The strategic process of M&A change management requires reconfiguring cultural structures and providing extensive training to align organizational goals and resolve employee concerns [33].

During the changing phase, organizations undergo significant structural and operational adjustments due to M&A. Training programs are essential for equipping employees with the skills, knowledge, and competencies needed to adapt to new processes, technologies, policies, and job roles. The intersection of technological innovation and M&A underscores the pivotal role of HR practices in promoting development, innovation, and job satisfaction through effective training and executive support [34]. This emphasis on HR practices can empower organizational leaders and HR professionals.

#### Leadership.

Finally, in the refreezing stage, ongoing reinforcement through advanced training opportunities and integration into performance evaluations, where the new behaviors and practices are assessed and rewarded, helps solidify the new behaviors and practices in the organizational culture [24].

In Lewin’s Change Model, the refreezing stage is crucial for stabilizing and integrating changes into the organizational culture to ensure they become the new norm. Leadership support during this phase is essential to reinforce the change and prevent regression to old behaviors. Maintaining proper interrelationships and building a strong team spirit through a positive working environment can solve employees’ issues and boost their morale. A healthy working environment and strong relationships can make employees happy in any organization. Thus, leadership also plays a crucial role at this level ensuring success by influencing employee morale and integration processes [35].

Leadership plays a pivotal role in refreezing by providing ongoing support, communicating the benefits of the change, and aligning organizational systems and structures to support the new way of operating. Leaders can achieve this through consistent messaging, celebrating successes. Addressing resistance or barriers to change is another key role of leadership, ensuring that the change process is smooth and effective [24].

Research underscores the significant influence of leadership on M&A performance. Effective leadership is particularly crucial for multinational firms in emerging economies, especially as M&A activity rises. The concept of distributed leadership, which is gaining popularity, has the potential to significantly enhance the success of cross-border M&A. The model also highlights the importance of promoting knowledge transfer and interaction among acquiring and target business personnel is mediated by socialization integration mechanisms in this model. Furthermore, it suggests that the autonomy of the acquired firm may moderate the relationship between distributed leadership and M&A success. This paradigm not only helps explain cross-border M&A integration but also holds relevance for multinational firms in both developed and emerging nations, offering a promising outlook for the future of M&A [36].

M&A CEOs’ leadership approaches are focused on traits and styles that effectively influence employees. The study aims to identify the best leadership style for employee adaptation and alignment with post-M&A organizational culture. A key finding is the importance of"4I" dimension of inspirational motivation in transformational leadership, which has been shown to significantly enhance employee engagement and commitment during M&A. This finding is crucial as it reduces post-M&A people-organization fit issues enhances overall expertise in M&A leadership dynamics [37].

Sarasvathy’s effectuation theory-based case study revealed that M&A success tactics. The study examines how business leaders execute M&As in a global healthcare company. Eight experienced M&A-involved firm presidents provided data. The examination stressed leadership, value generation, connection building, and organizational governance. This study underscores the crucial role of leadership in M&A performance, making the audience feel the significance of the research [38].

M&A is a deliberate reaction to commercial and regulatory challenges. Hospitals focus more on M&A for community health and the US economy [39]. This study examines the complex relationship between IT integration and senior leadership involvement in 72 Midwestern hospitals after M&A. Senior leadership involvement is statistically significant for M&A integration success. According to the study, senior leaders should actively participate in integration to handle M&A complexity.

The study meticulously examines the impact of transformative leadership and TMT heterogeneity on Chinese M&A performance. By identifying leadership-related M&A integration elements in China it fills a significant knowledge gap. The theoretical framework that connects leadership, TMT heterogeneity, and M&A outcomes is thoroughly explored across six Chinese industries with 295 respondents. The findings highlight the substantial influence of transformative leadership strongly affects M&A integration. Furthermore, the positive correlation between TMT heterogeneity and integration degree and speed underscores the crucial role of diversity in leadership in moderating M&A integration and post-M&A success [40].

HR procedures significantly impact organizational ambidexterity in M&A, with distributed leadership playing a pivotal role as a mediator of HR practices that enhance performance outcomes. M&A activities, which are inherently collaborative, greatly benefit from shared goals under distributed leadership [41]. Conversely, another study identified vital elements influencing HR management success in international M&A implementation, highlighting the critical stages of leadership consolidation, HR provision and evaluation, culture assessment, and process control [42]. Effective leadership anticipates and resolves issues, fostering trust and motivation among personnel, thereby enhancing M&A implementation success.

#### Performance of M&A.

From 2014 to 2018, 138 M&A agreements were identified to examine how M&A transactions affect US and European companies’ return on sales. The study compares the return on sales to the equity-to-enterprise value ratio. Both regions considerably reduced post-transaction return on sales, with the US falling 6.8% and Europe -5.3%. Regression research shows no correlation between M&A and corporate performance. The study finds that target firm attractiveness increases return on sales, but M&A does not affect post-transaction performance. These findings are crucial in understanding the complex relationship between M&A and financial performance and they underscore the need for further study to improve financing techniques [43].

The 2004 Saradar Bank-Audi Bank financial services merger explored how this M&A event affected Audi-Saradar Group’s financial performance, concentrating on critical variables. Return on equity, return on assets, and income per share quantify M&A impact. Pre- and post-merger Audi-Saradar Group financial performance is compared (2000–2003, 2004–2007). A paired sample T-test and ratios compare financial indicator changes before and after the merger. Data showed higher returns on assets and equity. Importantly, non-significant improvements occurred, providing reassurance about the merger’s impact. The merger raised earnings per share, improving this financial indicator [44].

A study used numerous variables to examine Pakistani banks’ M&A success and how it affected financial performance. Financial metrics include profitability and leverage. The analysis uses accounting ratios, Thomson Financial Services Worldwide M&A database, bank website financial statements, and Pakistan stock exchange data. The report highlights the need to examine bank mergers’ financial effects due to the financial sector’s crucial role in the national economy and its incentives, such as market share, cost-cutting, synergies, and uncovering hidden values. According to the data, these mergers did not increase Pakistan’s banking sector’s profitability or leverage [45].

The authors of this study have extended their analysis to the M&A landscape in Indian and Chinese markets using mean and ordinary least squares. The findings of this analysis, which revealed that M&A does not necessarily enhance business values in these countries, contribute to the global M&A literature. The study has enriched the understanding of M&A literature in underdeveloped economies in three distinct ways. It also sheds light on the dearth of empirical studies on M&A-related abnormal return changes. The study’s innovative approach to comparing M&A-generated anomalous returns with firm-specific characteristics is a valuable addition to the academic discourse, helping researchers overcome M&A announcement shortcomings. The study concludes that while M&A is still an emerging trend in developing countries it has the potential to foster growth and profitability [46].

Performance is a critical variable in the context of M&A, yet the process by which corporations ascertain the synergetic value of a target firm remains a mystery. The authors of this study argue that a comprehensive understanding of M&A synergies can significantly impact performance. To this end, the study employed a qualitative technique, conducting interviews with 50 50 M&A decision-makers and advisors from major corporations. These findings provide practical insights that can guide corporations in their M&A strategies [47].

Research examines how cross-border M&A affects profitability, leverage, sales, and capital growth. The authors sample 415 Italian bidding companies. The results demonstrate that cultural distance benefits bidder businesses post-M&A and hurts target companies. Acquiring size, experience, and cross-cultural management competencies affect cultural distance. Bidder businesses’ profitability ratios increased significantly post-M&A, while target companies did not. Cross-border M&A does not benefit the acquirer from the target company’s performance. M&A success depends on many things, including the state of the global economy, the economic conditions of the countries involved, and the industry-specific economic factors. M&A activity in Italy has improved due to consolidation, corporate repositioning, and internationalization [48].

Corporate governance, which refers to the system of rules, practices, and processes by which a company is directed and controlled, affects financial performance before and after M&A. The study explores how board size, independent commissioners, and ownership structure affect financial performance before and after M&A in 22 Indonesian stock exchange-listed companies from 2017 to 2018. Before M&A, independent commissioners increased ROE, while managerial ownership decreased it. Board size did not affect ROE. During M&A integration, good corporate governance’, which includes transparency, accountability, and fairness in decision-making, can boost financial performance. Good corporate governance improves company performance and value [49].

#### Conceptual framework.

**Fig 2:**
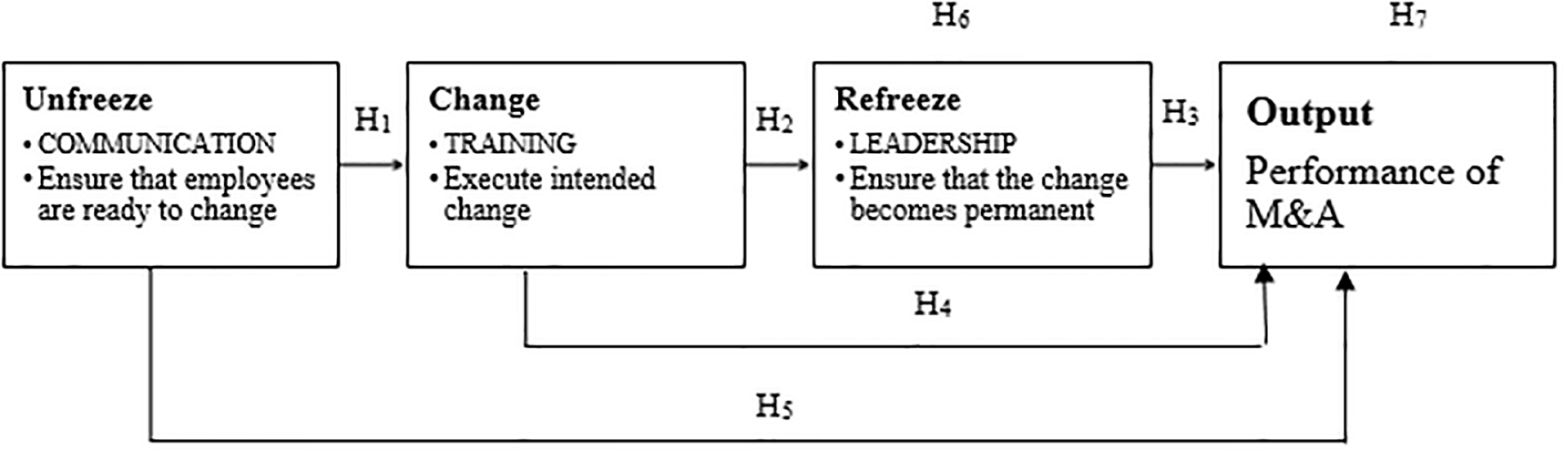
Proposed conceptual framework. Source: Authors’ Illustration.

Based on existing literature, a conceptual framework has been designed to examine the impact of human resource practices on the performance of M&A in the telecommunication industry in Sri Lanka, as illustrated in Fig 2. The framework, specifically tailored to the field of human resources and business management, aims to understand how specific HR practices influence the success of M&A activities, which is particularly relevant given the complex and often disruptive nature of mergers and acquisitions.

#### M&A performance is considered as a dependent variable.

This is the primary outcome of interest, reflecting how well the M&A activities achieve their intended objectives, such as improved operational efficiency, market expansion, or increased profitability. Independent variables are **communication, training, and leadership.** Effective communication is crucial during M&A processes as it helps manage employee expectations, a key factor in building confidence and trust in the process, reducing uncertainties, and fostering a positive organizational culture.

The framework was developed based on Lewin’s change model. **The first phase is unfrozen**, **a significant tool in the M&A process.** This stage involves preparing the organization for change by addressing the need for M&A and creating a sense of urgency. Effective communication is critical in this phase to ensure that employees understand the reasons behind the M&A and are ready to move away from the status quo. Clear communication ensures that employees are well-informed about the changes, which can mitigate resistance and promote smoother integration.

#### The second phase is change.

During this phase, the organization transitions to new working methods. Training programs play a critical role by empowering employees with the necessary skills and knowledge to operate within the new organizational framework. Training programs are essential for equipping employees with the necessary skills and knowledge to adapt to new systems, processes, and organizational structures that result from M&A activities. Proper training can enhance employee competence and confidence, leading to better performance and reduced errors during the transition period.

#### The final phase is refrozen.

This final stage involves solidifying the new changes to make them a permanent part of the organization. Strong leadership is essential to reinforce the new behaviors and practices, ensuring the changes are sustained over time. Continuous communication and follow-up training can help in embedding the new culture and operational practices. Strong leadership is vital in guiding employees through the M&A process. Effective leaders can inspire trust, provide direction, and support employees during the transition, ensuring that the organizational changes are implemented successfully, and employees remain engaged and motivated.

By focusing on these HR practices and aligning them with Lewin’s change model, the conceptual framework aims to provide a comprehensive understanding of how to effectively manage the human aspects of M&A in the telecommunication industry in Sri Lanka, ultimately improving M&A performance.

Based on the reviewed literature the following hypotheses are developed.

**H**_**1**_: Communication significantly impacts training.
**H**_**2**_: Training significantly impacts leadership.
**H**_**3**_: Leadership significantly impacts on performance of M&A.
**H**_**4**_: Training significantly impacts on performance of M&A.
**H**_**5**_: Communication significantly impacts on Performance of M&A.
**H**_**6**_: Training significantly impacts leadership mediated with communication.
**H**_**7**_: Communication, training and leadership significantly impact on performance of M&A.

## Materials and methods

### Research design, approach, and strategy

This study employs a deductive approach, aligning with a quantitative research design, to examine the impact of human resource practices on M&A performance in the telecommunication industry in Sri Lanka. The study focuses on executive-level employees from four leading telecommunications companies in Sri Lanka who have experienced M&A. The sample size, determined using the Morgan sampling table, was calculated with a 95% confidence level, a 5% margin of error, assuming a population proportion of 50%, and considering an unknown population. Accordingly, data was collected from 384 respondents who were selected based on the Morgan sampling table from May 2023 to August 2023 [50]. In practice, 322 responses were received, representing a response rate of 83.8%. Data cleaning is essential in research to ensure data quality and reliability by identifying and correcting missing or incorrect data and verifying responses for completeness and consistency. In this study, 322 responses were initially obtained. After removing missing and incorrect responses, 201 was deemed valid for data analysis.

### Survey instrument and data cleaning

The questionnaire, a crucial part of the research, consisted of two sections: Section A gathered demographic data, and Section B focused on three independent variables—employee training, communication, and leadership—and the dependent variable, M&A performance. Questions were carefully crafted for clarity and relevance, beginning with company affiliation and followed by variable-related perceptions. A five-point Likert scale from "Strongly Disagree" to "Strongly Agree" was used to ensure uniformity and comparability. The questionnaire underwent rigorous pilot testing with 50 respondents, validating its clarity and effectiveness, and was refined before distribution to the target sample. This validation process reassures the audience about the robustness of the study’s methodology and their integral part in it.

### Data analysis

Descriptive statistics were employed to compute the mean and standard deviation of agreement levels, providing measures of average value and variability. The study used the powerful analytical tool, the Structural Equation Model (SEM), to identify significant factors influencing the three phases of the change management model. All variables in this study were latent and could not be directly measured. SEM, a set of multivariate statistical procedures, determined and assessed causation, offering robust and reliable insights into the relationships among variables [51]. The measurement model, depicted in Eq 1, encapsulates the entire SEM framework, highlighting intricate connections and dependencies [52]. The use of SEM and the comprehensive data analysis process in SPSS AMOS provide the audience with confidence in the study’s findings.


$$\text{y}=\Lambda_{\text{y}}\left(\text{B}\eta +\zeta \right)+\epsilon$$ (1)

where: y = vector of observed variables; Λy = matrix of factor loadings relating observed variables to latent variables; B = matrix of structural coefficients among latent variables; η = vector of latent variables; ζ = vector of structural disturbances; ϵ = vector of measurement errors.

### Ethical consideration

The study, conducted with utmost respect for participants’ rights, adhered to stringent ethical standards. It is important to note that participation was entirely voluntary, and participants were fully informed about the study’s nature, purposes, procedures, potential risks, and benefits before agreeing to participate. Both written and verbal consent were obtained, with a consent statement included in the questionnaire, which participants had to acknowledge and agree to before proceeding.

Confidentiality and anonymity were rigorously maintained throughout the research process. Identifiable information was anonymized to protect participant identity, and all data were securely stored in password-protected files accessible only to the research team who were trained in data protection and privacy. This approach ensured that participants’ privacy was safeguarded at all stages of the research.

The research proposal received formal approval from the SLIIT Business School Research Committee under the designation SLIIT/ERC/SBS/2023/13. The study also adhered to the guidelines set forth by the Charter of the SLIIT Ethics Review Committee, ensuring that all ethical considerations were thoroughly addressed. Importantly, ethical conduct was continuously monitored throughout the study, demonstrating our unwavering commitment to maintaining high standards of research integrity.

To protect participants’ rights and dignity, the study refrained from using financial incentives or coercion to induce participation. It is important to reiterate that participants were assured that their involvement was voluntary and that they could withdraw from the study at any point without any negative consequences.

The findings were reported with the utmost honesty and transparency, contributing to a broader understanding of mergers and acquisition processes while respecting the rights and dignity of all participants. By adhering to these ethical principles, the study ensured that valuable insights were gained, and the audience can be confident in the findings, while upholding the highest standards of ethical research practice.

## Results and discussion

### Descriptive statistics

Table 1 summarizes descriptive statistics for four variables: communication, employee training, leadership, and M&A performance. The mean rating for employee training is around 3.37, with a standard deviation of about 0.99, suggesting that employee training is close to 4 on average. The mean rating for communication is approximately 3.84, with a standard deviation of around 0.63. These results indicate that communication scores are close to 4 on average. The mean leadership rating is 3.73, with a standard deviation of 0.73, suggesting that leadership scores, on average, are close to 4. The mean performance rating is 3.44 with a standard deviation of 0.86; on average performance scores are close to 4. In summary, the mean for all the variables is close to 4, indicating a high level of agreement among the respondents, which should instill confidence in the survey results.

**Table 1:** Descriptive statistics for four variables.

Variable	Mean	Std. Deviation	N
Performance	3.44	0.86	201
Communication	3.84	0.62	201
Employee Training	3.37	0.99	201
Leadership	3.73	0.73	201

Source: Researchers created.

### Factor analysis

Table 2 presents an analysis that examines the reliability and validity of four key constructs—Communication, Training, Leadership, and Performance—through a variety of statistical measures, including Factor Loadings, Kaiser-Meyer-Olkin (KMO) Measure, Average Variance Extracted (AVE), Composite Reliability (CR), Cronbach’s Alpha, and Variance Inflation Factor (VIF).

**Table 2:** Factor analysis.

Variable		Factor Loading (> 0.5)*	KMO (> 0.6)*	AVE (> 0.5)*	CR (> 0.5)*	Cronbach’s alpha (> 0.7)**	VIF (> 0.1)*
Communication	C1	.681	0.911	0.69	0.89	0.984	3.325
	C2	.718					
	C3	.678					
	C4	.663					
	C5	.679					
	C6	.721					
	C7	.674					
	C8	.706					
	C9	.685					
Training	T1	.656	0.914	0.664	0.877	0.979	3.408
	T2	.730					
	T3	.514					
	T4	.725					
	T5	.680					
	T6	.695					
	T7	.638					
	T8	.673					
	T9	.675					
Leadership	L1	.735	0.721	0.713	0.757	0.922	2.605
	L2	.663					
	L3	.742					
Performance	P1	.736	0.893	0.701	0.854	0.977	----
	P2	.677					
	P3	.660					
	P4	.648					
	P5	.758					
	P6	.732					
	*Extraction Method*: *Principal Component Analysis*.						
	*a*. *4 components extracted*.						
	*Bartlett’s test of Sphericity (Sig) = (Sig < 0*.05)*						

Source: Researchers created; Shrestha [54]*; [52]**

### Sample adequacy

The KMO measure, which assesses the adequacy of the sample for factor analysis, is above 0.6 for all constructs [54]. Particularly, Communication (0.911) and Training (0.914) exhibit excellent sample adequacy, which strengthens the reliability of the factor analysis, while Leadership (0.721) and Performance (0.893) also show acceptable levels of sample adequacy. This indicates that the sampling is sufficient for extracting meaningful factors.

### Item-construct association

The SEM approach began with a thorough confirmatory factor analysis. Varimax rotation was used to extract and minimize factors. According to the Eigenvalue criterion, only factors with Eigenvalues greater than one were chosen. The factor loadings for all variables surpass the recommended threshold of 0.5, indicating that each item is sufficiently suitable for SEM analysis [54]. Specifically, the factors loadings for Communication range from 0.663 to 0.721, suggesting that the items within these constructs are strongly representative of the overall factor. Similarly, Training exhibits loadings between 0.514 and 0.730, Leadership shows loadings between 0.663 and 0.742, and Performance ranges from 0.648 to 0.758. These values collectively confirm that the constructs are effectively capturing the intended dimensions.

### Convergent validity

Furthermore, the Average Variance Extracted (AVE) for each construct is greater than 0.5, confirming good convergent validity [54]. AVE reflects the proportion of variance explained by a latent construct in relation to the total variance in its items. Leadership, with an AVE of 0.713, has the highest explained variance, which suggests it is the strongest predictor among the constructs. Communication (0.69) and Training (0.664) also show robust convergent validity, while Performance (0.701) remains within an acceptable range.

### Internal consistency

The Composite Reliability (CR), which evaluates the internal consistency of the items, similarly supports the reliability of the constructs. Communication (0.89), Training (0.877), and Performance (0.854) all exhibit high CR values, demonstrating strong consistency in their respective items. Even Leadership, with a CR of 0.757, remains above the acceptable threshold, further reinforcing the reliability of the scale [54].

### Reliability

To test the reliability Cronbach’s Alpha test was performed and findings are illustrated in Table 2. Cronbach’s alpha value for the variables, employee training is 0.979, communication is 0.984, leadership is 0.922, and M&A performance is 0.977. All these values fall above 0.9, indicating high internal consistency among the questions in the survey [52]. It suggests that the questions are highly reliable and consistently measure the same underlying construct.

### Multicollinearity

Multicollinearity was assessed using tolerance and the Variance Inflation Factor (VIF). Tolerance measures how much of the variance in one predictor is not explained by other predictors, whereas VIF is its reciprocal and indicates the degree of multicollinearity [54]. A VIF value greater than 1 suggests the presence of multicollinearity. In this study, the VIF for communication is 3.325, for employee training is 3.408, and for leadership, it is 2.605. These values indicate the presence of multicollinearity among the three independent variables (Table 2). However, since all VIF values are below 5, they suggest only moderate multicollinearity among the variables.

### Normality test

To validate the outcomes of the regression analysis, it was necessary to exhibit a normal distribution in the model residuals. The methodology employed entailed an evaluation of the histogram and normal probability to ascertain the normality of the model residuals. The regression standardised residual histograms in communication and training revealed that the data was normally distributed by displaying a bell-shaped curve in the histogram of the model residuals while, the histogram of leadership show that the curve slight skewness to right and the curve of the performance histograms are skewed slightly to right. From the Quantile-Quantile Plot, it can be said as normal if the points are near the diagonal line. The distribution of variables was observed to be skewed around the mean (S1 Fig). Therefore, the assumptions of normality were not found to have been violated.

#### Model fit.

Path Analysis is used to test the hypotheses using Structural Equation Modeling. Before testing the hypotheses, the following analysis is conducted to determine the goodness of fit of the model.

The confirmatory Factor Analysis (CFA) was instrumental in establishing the convergent and discriminant validity of the model. The results, detailed in Table 3, confirm the model’s Chi-square = 550.561, Degrees of freedom = 310 at the Probability level = .005. Chi-square/ Degree of Freedom (χ2/ d.f.) = 1.77 is supported. The Goodness-of-fit Index (GFI) = .846, though slightly below the accepted level, is acceptable given the sample size [55, 56]. The comparative Fit Index (CFI) = .926; Tucker Lewis Index (TLI) = .916; Root Mean Square Error of Approximation (RMSEA) = .062 and Root Mean Square Residual (RMR) = .031. These results confirm the model’s validity and fit for hypothesis testing.

**Table 3:** Model fitting analysis.

Index Name	Index	Model	Level of Acceptance		Fit Condition
**Absolute fit**	RMSEA	.062	<.08	[57]	Acceptable
**Comparative fit**	CFI	.926	≥.90	[58]	Acceptable
**Parsimonious fit**	Chi-sq./df	1.77	>3	[53]	Acceptable
**Incremental Fit**	IFI	.926	≥.90	[58]	Acceptable
**Tucker-Lewis Index**	TLI	.916	≥.90	[58]	Acceptable
**Goodness-of-fit Index**	GFI	.846	≥.90	[59]	Slightly lower
**Root Mean Square Residual**	RMR	.031	<0.05	[56]	Acceptable

Source: Authors’ representation based on AMOS results

### Results of the study

This study is unique in that it tests a comprehensive set of hypotheses related to training, communication, and leadership in the context of M&A performance. H_1_ was developed to test whether there is any significant direct relationship between training and communication, while H_2_ tests whether there is any significant direct relationship between leadership and training. Hypotheses three to five were developed to determine whether there is a direct relationship between the performance of M&A and communication (H_3_), training (H_4_), and leadership (H_5_), respectively. H_6_ tests whether training significantly impacts leadership mediated with communication, while H_7_ Performance of M&A significantly impacts communication mediated with training and leadership.

The analysis uncovers several significant relationships among the latent variables. Communication exerts a robust and significant positive effect on training, with a standardized estimate of β = 0.800, thereby confirming hypothesis H_1_. This finding is significant as it illuminates the crucial role of communication in the training process.

**Fig 3:**
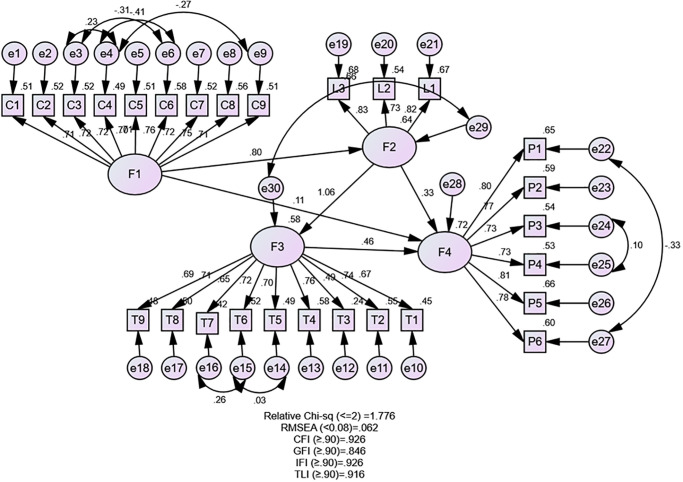
Path analysis. Source: Authors’ Illustration based on the AMOS results. Where F1 = Communication; F2 = Training; F3 = Leadership and F4 = Performance of M&A.

H_7_ was developed to test the direct effect of three variables, communication, training, and leadership, on the performance of M&A. The results showed training has a notable indirect effect (β = 0.487) H_1_. Communication, while not having an indirect effect on training, plays a significant role in influencing it, thereby maintaining the total effect remains equivalent to the direct effect. The training variable, with an R^2^ value of 0.64, is explained by 64% of these variables (Tables 4 and 5, Fig 3). The findings indicate that an employee’s readiness to change directly impacts on the execution of the intended change. According to the first phase of Lewin’s change model, individuals must be prepared to change; otherwise, the anticipated change may not be effectively implemented. Therefore, effective communication at the outset is crucial, as it directly influences training, which in turn facilitates employees’ gradual adaptation to new conditions.

**Table 4:** Hypothesis testing based on regression weights.

Relationship	β	P	Results
Training <--- Communication	.800	***	H_1_ Accept
Leadership <--- Training	1.062	***	H_2_ Accept
Performance <--- Leadership	.459	***	H_3_ Accept
Performance <--- Training	.332	.003	H_4_ Accept
Performance <--- Communication	.111	.352	H_5_ Rejected

Note(s):

***p < 0.001.

Source: Authors’ Illustration based on the AMOS results

**Table 5:** Direct effect, indirect effect and total effect of the variables.

Dependent Latent Variables	Independent Latent Variables	Standardized Estimates		
		Direct	Indirect	Total
Training H_1_ (R^2^ = 0.64)	Communication	.800	.000	.800
Leadership H_6_ (R^2^ = 0.58)	Training	1.062	.000	1.062
	Communication	.000	.850	.850
Performance H_7_ (R^2^ = 0.72)	Communication	.111	.655	.766
	Training	.332	.487	.819
	Leadership	.459	.000	.459

Source: Authors’ Illustration based on the AMOS results

The results demonstrate a significant direct relationship between training and leadership (β = 1.062, p < 0.000), supporting hypothesis H_2_. However, substantial indirect effects of communication through training (β = .850, p < 0.000), contributing to its total effect on communication on leadership. This finding, which supports H6, adds a layer of complexity to our understanding of the relationship between communication, training, and leadership. The leadership variable with an R^2^ value of 0.58 is explained by these variables (Tables 4 and 5, Fig 3). According to Lewin’s change model, there is a connection between the phases of unfreezing, changing, and refreezing. The results also indicate that the second phase of Lewin’s model is supported. Effective communication aids in convincing and preparing individuals for change, while leadership plays a crucial role in supporting the implementation of these changes.

As depicted in Tables 4 and 5, and Fig 3, leadership exerts a moderate direct effect on performance with a standardized estimate of β = 0.459, p < 0.000, which supports hypothesis H_3_. This finding highlights the significant role of leadership in the M&A process.

The analysis of Performance (H_7_, R^2^ = 0.72) reveals the direct, indirect, and total effects of communication, training, and leadership on performance. Communication has a direct effect of 0.111, an indirect effect of 0.655, and a total effect of 0.766, indicating a moderate overall influence but a relatively small direct impact on performance. Training shows a direct effect of 0.332, an indirect effect of 0.487, and a total effect of 0.819, demonstrating a stronger combined influence on performance. Leadership, on the other hand, has a direct and total effect of 0.459 with no indirect effects, underscoring its significant direct role in driving performance. This intriguing finding engages us in understanding the complex dynamics of M&A performance, which has the highest R^2^ value of 0.72, is influenced by communication, training, and leadership directly and indirectly. These findings highlight the crucial role of communication in enhancing training and of training in improving both leadership and performance, while leadership itself also significantly contributes to better performance. The R^2^ value, a measure of how well the model explains the variance in the dependent variable, indicates that the model effectively explains 72% of the variance in performance, showing that the model is reasonably effective in explaining the relationships among these latent variables.

Thus, the conceptual model results indicated that communication significantly influences training, leadership is highly influenced by training and indirectly by communication, and performance is influenced by all three variables—communication, training, and leadership—through both direct and indirect pathways. The R^2^ values indicate that the model effectively explains 64% of the variance in training, 58% of the variance in leadership, and 72% in performance, showing that the model is reasonably effective in explaining the relationships among these latent variables.

## Discussion

The analysis reveals that communication significantly affects training, with a standardized estimate of β = 0.800. This finding underscores the critical role of communication in the training process and aligns with existing literature on organizational change. Various previous studies support the results of this study.

Effective communication at the outset of change initiatives is crucial, as it directly influences training, facilitating employees’ gradual adaptation to new conditions. This is consistent with the first phase of Lewin’s change model, where individuals must be prepared for change for the anticipated transformation to be effectively implemented [60]. Studies have shown that clear and consistent communication helps reduce uncertainty and resistance among employees, thereby enhancing their readiness to embrace change [24]. This reassurance is vital in reducing apprehension and fostering a positive attitude towards change, providing the audience with practical tools to navigate organizational change.

Moreover, the analysis highlights that an employee’s readiness to change significantly impacts the execution of the intended change. This aligns with findings from recent research that emphasize the importance of psychological readiness and its role in successful change management [61]. The results of this study, therefore, make a significant contribution to the broader understanding of the dynamics between communication, training, and change readiness in organizational settings.

The findings underscore the necessity of robust communication strategies in supporting training initiatives during organizational change [62]. By ensuring employees are well informed and prepared, organizations can enhance their adaptability and successfully implement change [63]. Communication not only assists learners in understanding objectives, procedures, and expectations, but also significantly improves results. Effective communication is the key to improving training, understanding, and recall. The study indicated that learners were likelier to understand and implement training content when instructors used clear, succinct language and allowed questions and feedback [25].

The analysis reveals a significant direct relationship between training and leadership (β = 1.062, p < 0.000). This finding highlights the crucial role of training in leadership development. Additionally, communication has a substantial indirect effect on leadership through training (β = .850, p < 0.000), indicating that the total effect of communication on leadership is significant. Previous research findings support these findings.

According to Lewin’s change model, the phases of unfreezing, changing, and refreezing are interconnected, forming a continuous change management cycle. The findings indicate that effective communication is essential in the unfreezing phase, where it plays a powerful role in convincing and preparing individuals for change. This preparation is critical as it sets the stage for the subsequent phases of change and implementation [60]. Clear and transparent communication ensures that employees understand the need for change, its benefits, and the steps involved, thereby reducing resistance and increasing readiness for change.

Training plays a pivotal role in the changing phase of Lewin’s model by providing employees with the necessary skills, knowledge, and competencies to adapt to new processes and systems. This is consistent with the first phase of Lewin’s change model, where individuals must be prepared for change for the anticipated transformation to be effectively implemented. The significant direct relationship between training and leadership highlights the importance of equipping leaders with the capabilities to guide and support their teams through the change process, making them feel valued and integral to the change process. Leaders who have undergone comprehensive training are better prepared to address challenges, provide clear direction, and motivate their teams, leading to more effective change implementation [63, 64]. Effective leadership role strengthens organizational change [65].

Furthermore, the substantial indirect effect of communication on leadership through training underscores the importance of an integrated approach to change management. Effective communication prepares individuals for change and enhances the impact of training programs by ensuring that the information conveyed is understood and retained. This, in turn, empowers leaders to execute their roles more effectively. This empowerment fosters a supportive environment for change, instilling a sense of confidence and security in the audience [24, 66].

In the refreezing phase, leadership plays a crucial role in reinforcing new behaviors and practices, ensuring the changes become embedded within the organizational culture. Leaders who have been effectively trained and supported by clear communication are more likely to sustain the momentum of change, address any residual resistance, and institutionalize the new norms and practices [67]. This continuous cycle of communication, training, and leadership is not just important, but it is essential for achieving successful and sustainable organizational changes, and it requires the commitment and engagement of all involved.

The findings revealed that leadership exerts a moderate direct effect on performance (β = 0.459). This finding aligns with existing literature, which emphasizes the critical role of leadership in driving organizational performance, particularly during periods of significant change such as M&A. Effective leadership is crucial for setting a clear vision, motivating employees, and ensuring alignment of goals across the organization [68, 69]. Leaders who can navigate the complexities of M&A there by enhancing performance by fostering a positive organizational culture and effectively managing resources, provide a reassuring beacon of stability in times of change [70]. This emphasis on the role of leadership can inspire and motivate the audience.

Moreover, the findings show that training significantly positively affects performance (β = 0.332). Training equips employees with the necessary skills and knowledge to adapt to new processes and systems introduced during M&A, thereby enhancing their performance. Literature underscores the importance of continuous learning and development in improving organizational outcomes, inspiring a culture of growth and improvement [25, 71]. Effective training programs can mitigate the negative impacts of change, reduce uncertainty, and boost employee confidence and competence, leading to improved performance [72].

However, the effect of communication on performance is insignificant (β = 0.111; p-value of 0.352). This finding suggests that while communication is essential for preparing and informing employees about change, its direct impact on performance may be limited. The literature suggests that communication alone may not suffice to drive performance improvements; it must be coupled with other supportive measures, such as leadership and training, to have a meaningful impact [73]. Effective communication is necessary to ensure clarity and reduce resistance to change, but it may not directly translate into performance gains without the accompanying support structures.

Hypothesis H_7_ was developed to test the direct effects of communication, training, and leadership on the performance of M&A. The findings underscore the pivotal role of training, leadership, and communication in influencing the performance of M&A. Training has a strong total effect of β = 0.819, and leadership has a direct effect (β = 0.459) on performance. Although communication does not directly affect performance, it has a significant indirect effect (β = 0.655). This indirect effect of communication on performance is significant because it highlights the complex and nuanced relationship between communication, training, and leadership, and how they collectively shape the context in which performance is achieved.

Training plays a pivotal role in M&A performance, emphasizing its critical role in equipping employees with the skills and knowledge necessary to navigate the complexities of M&A. Well-designed and implemented training programs can significantly enhance employees’ abilities to adapt to new processes and organizational changes. Effective training interventions during M&A processes can reduce employee resistance, enhance engagement, and improve overall performance by providing clarity and building competence [28]. There is a strong correlation between training efficacy and employee commitment to the newly consolidated organization [74], underscoring the importance of training in successful M&A processes.

Leadership’s direct effect on performance highlights the importance of strong, transformational leaders in the M&A process. Effective leadership can drive the successful implementation of strategic changes and foster a positive organizational culture. Leaders who can inspire, motivate, and guide their teams can significantly impact the success of M&A initiatives. Leadership during M&A is crucial for maintaining employee morale, ensuring alignment with new strategic goals, and navigating the complexities of organizational integration [38, 75]. This underscores the importance of direction and clarity in leadership, making employees feel secure and focused.

Findings revealed that communication does not have a direct effect on performance. However, its significant indirect effect through training and leadership underscores its essential role in facilitating successful M&A outcomes. Clear and consistent communication is vital for reducing uncertainty, building trust, and ensuring that employees are well-informed about changes. Effective communication can enhance the impact of training programs by providing context and clarity, thereby improving employees’ understanding and acceptance of new practices. Transparent communication strategies can significantly enhance the effectiveness of training and leadership during organizational changes [18, 76].

The substantial total effects of training and communication on performance, mediated through leadership, underscore the necessity of a comprehensive approach to managing M&A is necessary. This approach involves integrating robust training programs, strong leadership, and effective communication strategies to drive successful outcomes. The interplay between these variables highlights the importance of a multi-faceted strategy that addresses various aspects of organizational change during M&A. Recent literature emphasizes that a holistic approach to M&A management, including training, leadership, and communication, can lead to better integration and performance outcomes [77].

## Conclusion

This study has explored the relationship between employee training, communication, and leadership in the context of M&A performance in the telecommunications industry in Sri Lanka. The study’s findings highlight the practical implications of these relationships, providing valuable insights into the factors that drive the effectiveness of changes. The study underscores the complex and diverse factors that influence performance during mergers and acquisitions. Communication has a strong positive effect on training (β = 0.800), contributing to 64% of the variance in training. Training significantly influences leadership (β = 1.062), accounting for 58% of the variance in leadership. Both leadership (β = 0.459) and training (β = 0.332) have significant positive impacts on performance, which explains 72% of the variance in performance. However, communication does not have a significant direct effect on performance (β = 0.111). Overall, the results underscore the importance of communication, training, and leadership in driving performance, particularly in the context of mergers and acquisitions. These findings stress the need for a comprehensive approach that integrates these fundamental components to enhance the performance and adaptability of an organization in a constantly changing business environment. To optimize performance during M&A, organizations should prioritize the development of robust leadership skills and invest in employee training, ensuring that communication is aligned with these efforts. By applying the principles of Lewin’s change model, organizations can effectively manage change in a structured and practical manner.

### Theoretical implications

This study reinforces Lewin’s change model by demonstrating its applicability in the context of M&A in the telecommunications industry. It shows that the model’s structured approach to managing change is effective, thus contributing to the theoretical understanding of change management processes. Moreover, findings highlight the interconnectedness of communication, training, and leadership in influencing organizational performance during M&A. This suggests that these factors should not be studied in isolation but rather in a holistic view of change management, which considers their interdependence and underscores the need for a comprehensive strategy. This enriches the theoretical framework by promoting a more integrated approach to change management. The finding that communication has an indirect influence on performance rather than a direct one adds nuance to existing theories. It suggests that communication might be more effective as a facilitator for leadership and training rather than as a standalone factor, prompting a re-evaluation of its role in theoretical models of organizational change.

### Practical implications

Organizations should invest in leadership development programs to cultivate strong leaders who can effectively guide the company through M&A processes. Effective leadership is crucial for navigating the complexities of M&A and ensuring successful integration and performance. Allocating resources toward comprehensive employee training programs is vital. Emphasizing the role of well-trained employees in enhancing overall performance during M&A will make the audience feel confident in their team’s capabilities. Well-trained employees are better equipped to adapt to new processes and systems, which can enhance overall performance during M&A. Organizations should develop communication strategies that support and enhance these efforts, ensuring that all stakeholders are informed and engaged throughout the M&A process. The findings underscore the importance of strategic resource allocation. Organizations should ensure that adequate resources are dedicated to leadership development, employee training, and communication to support successful M&A outcomes. Companies should adopt a holistic approach to change management, recognizing the interdependence of leadership, training, and communication. This involves creating a cohesive strategy that leverages these elements to improve organizational flexibility and performance. By integrating these practical insights, organizations can enhance their ability to manage change effectively, particularly in the dynamic and competitive telecommunications industry, making the audience feel equipped and prepared for the challenges of M&A.

### Limitations

While this study has provided valuable insights into the relationship between employee training, communication, leadership, and M&A performance within the telecommunication industry in Sri Lanka, it is essential to acknowledge that there are some limitations that may affect the generalizability of the findings. Firstly, this research is limited to the context of the telecommunication industry in Sri Lanka, which means that there is a geographic concentration. The sample size of this study is relatively small, drawn from a specific geographic area and specific industry, which could limit the broader applicability of the results. Furthermore, the study focused on three independent variables, while real-world M&A scenarios often involve complex interactions. Finally, this study was conducted in a limited time frame, which may not capture longer-term impacts. Acknowledging these limitations underscores the need for further exploration in the field, encouraging the audience to continue the research.

## Supporting information

S1 Fig Results of the normality test. (TIF)

## References

[1] Shaengchart Y, Kraiwanit T, Virunhaphol S, Chutipat V, Chaisiripaibool S. Users’ opinions on telecom mergers and acquisitions in a developing country. Corporate & Business Strategy Review. 2023;4(1). https://ssrn.com/abstract=4338807

[2] Yahiaoui D, Chebbi H, Weber Y. HR practices, context and knowledge transfer in M&A. The International Journal of Human Resource Management. 2016;27(20):2415–35. doi: 10.1080/09585192.2016.1226192

[3] Inuk I. Examining The Impact Of Culture As A Factor In Mergers And Acquisitions: Time Warner & Aol’s Failed Merger. 2019. https://www.linkedin.com/pulse/examining-impact-culture-factor-mergers-acquisitions-time-inuk/?articleId=6560992343506464768

[4] Patel K. Top 11 Failed Mergers and Acquisitions of All Time. n.d. https://www.mascience.com/basics/top-11-failed-mergers-and-acquisitions-of-all-time

[5] Kim Y. Towards the Asian information society through telecommunications trade and investment. Global Economic Review. 1999;28(3):3–0. doi: 10.1080/12265089908449764

[6] LBO (Lanka business online). Sri Lanka Telecom & Mobitel join forces making history in national connectivity. 2021. https://www.lankabusinessonline.com/sri-lanka-telecom-mobitel-join-forces-making-history-in-national-connectivity/

[7] Lye RM. Review of Mergers & Acquisition Cases in Sri Lanka. Sri Lanka Journal of Marketing. 2022 Aug 8;8.

[8] Daily Mirror-online. Mobitel acquires 87.6% of E-Channeling as voluntary offer closes. 2016. https://www.dailymirror.lk/business-news/Mobitel-acquires-of-E-Channelling-as-voluntary-offer-closes/273-117034

[9] LBO (Lanka business online). Interview: Our aim is to provide a better 4G experience, Hutch CEO 2020. https://www.lankabusinessonline.com/interview-our-aim-is-to-provide-a-better-4g-experience-hutch-ceo/

[10] Jayasinghe BE, Rifat F. The effect of cross-border mergers & acquisitions as a driver of better corporate sustainability practices-A study on the post-acquisition context of selected target firms from Sri Lanka and Sweden. 2023. https://hdl.handle.net/2077/77839

[11] Wickramasinghe V, Sajeewani N. Mergers and acquisitions and employees’ level of anxiety: the role of HRM practices. International Journal of Happiness and Development. 2022;7(3):265–90. doi: 10.1504/IJHD.2022.127634

[12] Lewin K. Frontiers in group dynamics: Concept, method and reality in social science; social equilibria and social change. *Human Relations*, 1947. 1(1): 5–41. doi: 10.1177/001872674700100103

[13] Rajapakshe W. Driving Organizational Change In The Midst Of The Crisis: How Does It Affect Employee Performance?. 2021;10 (1). Available at: https://www.researchgate.net/publication/348676602_Driving_Organizational_Change_In_The_Midst_Of_The_Crisis_How_Does_It_Affect_Employee_Performance

[14] Kaur H. Is the Human Resources Function Essential in Achieving Successful Organisational Change?. 2021: 13–17. https://www.theseus.fi/handle/10024/564737

[15] Eliyana A. and Ma’arif S. Job satisfaction and organizational commitment effect in the transformational leadership towards employee performance. European Research on Management and Business Economics, 2019. 25 (3), 144–150. doi: 10.1016/j.iedeen.2019.05.001

[16] Angwin DN, Mellahi K, Gomes E, Peter E. How communication approaches impact mergers and acquisitions outcomes. The International Journal of Human Resource Management. 2016 Nov 12;27(20):2370–97. doi: 10.1080/09585192.2014.985330

[17] Wang J, Schweizer L. Chinese cross‐border mergers and acquisitions: How communication practices impact integration outcomes. Thunderbird International Business Review. 2024;66(1):81–99. doi: 10.1002/tie.22363

[18] Zagelmeyer S, Sinkovics RR, Sinkovics N, Kusstatscher V. Exploring the link between management communication and emotions in mergers and acquisitions. Canadian Journal of Administrative Sciences/Revue Canadienne des Sciences de l’Administration. 2018;35(1):93–106. doi: 10.1002/cjas.1382

[19] De Leon MV. Impact of managerial communication, managerial support, and organizational culture difference on turnover intention: A tale of two merged banks. *Problems and Perspectives in Management*, 2020; 18(4): 376–387. doi: 10.21511/ppm.18(4).2020.30

[20] Kucaladevi AN, Hernando S, Fathurokhman DT, Abdullah TM. Correlation of Communication and Compensation and Benefits on Employees Performance Mediated by Motivation Research on Companies Merger and Acquisition in Indonesia in Oil and Natural Gas Industry. Journal of Business and Management Studies. 2021;3(2):173–84. doi: 10.32996/jbms.2021.3.2.18

[21] Mathur M. Cross-border Merger and Acquisitions: The impact of tonality and effective communication on performance for acquiring firms. 2023. https://lup.lub.lu.se/luur/download?func=downloadFile&recordOId=9132189&fileOId=9132341

[22] Malmmose M, Lueg R. Business model communication and financial performance in cross national acquisitions. Journal of Business Models. 2019;7(5):70–89. doi: 10.5278/ojs.jbm.v7i5.3358

[23] Cummings TG, Worley CG. Organization development & change. 2016. *Organization Development and Change*. Cengage Learning.

[24] Hiatt JM, Creasey TJ. Change management: The people side of change. Prosci; 2003.

[25] Salas E, Tannenbaum SI, Kraiger K, Smith-Jentsch KA. The science of training and development in organizations: What matters in practice. Psychological science in the public interest. 2012;13(2):74–101. doi: 10.1177/1529100612436661 26173283

[26] Society for Human Resource Management (SHRM). The Importance of Effective Communication in the Workplace. 2014. SHRM website. https://www.shrm.org/topics-tools/tools/toolkits/managing-organizational-communication

[27] Sarala RM, Junni P, Cooper CL, Tarba SY. A sociocultural perspective on knowledge transfer in mergers and acquisitions. Journal of management. 2016;42(5):1230–49. doi: 10.1177/0149206314530167

[28] Weber Y. Development and training at mergers and acquisitions. Procedia-Social and Behavioral Sciences. 2015 Dec 3;209:254–60. doi: 10.1016/j.sbspro.2015.11.229

[29] Denison DR, Ko I. Cultural due diligence in mergers and acquisitions. In Advances in mergers and acquisitions 2016:53–72. Emerald Group Publishing Limited.

[30] Rodríguez-Sánchez JL, Ortiz-de-Urbina-Criado M, Mora-Valentín EM. Thinking about people in mergers and acquisitions processes. International Journal of Manpower. 2019;40(4):643–57. doi: 10.1108/IJM-05-2018-0143

[31] Tarba SY, Cooke FL, Weber Y, Ahlstrom D, Cooper CL, Collings DG. Mergers and acquisitions in the global context: The role of human resource management. Journal of World Business. 2020;55(2):101048. doi: 10.1016/j.jwb.2019.101048

[32] Thakur B, Lamsal R. Post-merger impact on employee performance of commercial banks in Nepal. *International Journal of Finance and Commerce*, 2023; 5(1): 83–91. https://www.commercejournals.com/assets/archives/2023/vol5issue1/5009-535.pdf

[33] Kang E, Nantharath P, Hwang HJ. The strategic process of merger and acquisition (M&A) market using integrating change management. Journal of Distribution Science. 2020;18(6):57–62. doi: 10.15722/jds.18.6.202006.57

[34] Christofi M, Vrontis D, Thrassou A, Shams SR. Triggering technological innovation through cross-border mergers and acquisitions: A micro-foundational perspective. Technological Forecasting and Social Change. 2019;146:148–66. doi: 10.1016/j.techfore.2019.05.026

[35] Zhang J, Ahammad MF, Tarba S, Cooper CL, Glaister KW, Wang J. The effect of leadership style on talent retention during merger and acquisition integration: Evidence from China. The International Journal of Human Resource Management. 2015;26(7):1021–50. doi: 10.1080/09585192.2014.908316

[36] Khan Z, Rao-Nicholson R, Akhtar P, He S. Cross-border mergers and acquisitions of emerging economies’ multinational enterprises—The mediating role of socialization integration mechanisms for successful integration. Human Resource Management Review. 2021;31(3):100578. doi: 10.1016/j.hrmr.2016.12.003

[37] Perdhana MS, Sawitri DR, Chaerunissa G. A Phenomenological Investigation of Person Organizational Fit: Characteristics and Leadership Styles. *International Journal of Professional Business Review*. 2022; 7(5): 1–13. doi: 10.26668/businessreview/2022.v7i5.734

[38] Ben Jacob A. Leadership Strategies for Improving Mergers and Acquisitions Performance. 2020. Walden Dissertations and Doctoral Studies. 7913. https://scholarworks.waldenu.edu/dissertations/7913

[39] Tella F. Relationship between Information Technology Integration, Senior Leadership Involvement in Postmerger Integration, and Merger Performance. Walden University; 2020. https://www.proquest.com/openview/40b9d98fb657cc71d9d6aaad0edb4fa6/1?pq-origsite=gscholar&cbl=18750&diss=y

[40] Mao D. Effects of transformational leadership and TMT heterogeneity on M&A integration and performance in China. 2021. PhD thesis, University of Essex. https://repository.essex.ac.uk/30306/

[41] Rao-Nicholson R, Khan Z, Akhtar P, Tarba SY. The contingent role of distributed leadership in the relationship between HR practices and organizational ambidexterity in the cross-border M&As of emerging market multinationals. The International Journal of Human Resource Management. 2020;31(2):232–53. doi: 10.1080/09585192.2016.1216882

[42] Rodríguez-Sánchez JL, Mora-Valentín EM, Ortiz-de-Urbina-Criado M. Successful human resources management factors in international mergers and acquisitions. Administrative Sciences. 2018;8(3):45. doi: 10.3390/admsci8030045

[43] Borodin A, Sayabek ZS, Islyam G, Panaedova G. Impact of mergers and acquisitions on companies’ financial performance. Journal of International Studies. 2020;13(2). https://www.ceeol.com/search/article-detail?id=979600

[44] Sujud H, Hachem B. Effect of mergers and acquisitions on performance of Lebanese banks. International research journal of finance and economics. 2018;166(2):69–77. https://www.internationalresearchjournaloffinanceandeconomics.com/

[45] Ahmed F, Manwani A, Ahmed S. Merger & acquisition strategy for growth, improved performance and survival in the financial sector. Jurnal Perspektif Pembiayaan Dan Pembangunan Daerah. 2018;5(4):196–214. doi: 10.22437/ppd.v5i4.5010

[46] Reddy K, Qamar M, Yahanpath N. Do mergers and acquisitions create value? The post-M&A performance of acquiring firms in China and India. Studies in Economics and Finance. 2019;36(2):240–64. doi: 10.1108/SEF-01-2018-0027

[47] Bauer F, Friesl M. Synergy evaluation in mergers and acquisitions: an attention‐based view. Journal of Management Studies. 2024;61(1):37–68. doi: 10.1111/joms.12804

[48] Cioli V, Giannozzi A, Ippoliti V, Roggi O. Cross-Border M&A and Financial Performance: Empirical Evidence on Bidder/Target Companies. International Journal of Business and Management. 2021;15(4):1–67. https://ideas.repec.org/a/ibn/ijbmjn/v15y2021i4p67.html

[49] Hastuti H, Selarasati D, Gunawan A, Muhammad R. The Effect of Corporate Governance on Financial Performance before After Mergers and Acquisitions. 2023; 6(7). https://www.ijassjournal.com/2023/V6I7/4146663391.pdf

[50] Krejcie RV, Morgan DW. Determining sample size for research activities. Educational and psychological measurement. 1970; 30(3):607–10. doi: 10.1177/001316447003000308

[51] Mueller RO, Hancock GR. Structural equation modeling. In The reviewer’s guide to quantitative methods in the social sciences. Routledge. 2018; 445–456.

[52] Stein CM, Morris NJ, Nock NL. Structural equation modeling. Statistical human genetics: Methods and protocols. 2012:495–512. doi: 10.1007/978-1-61779-555-8_27 22307716

[53] Straub DW. Validating instruments in MIS research. MIS quarterly. 1989:147–69. doi: 10.2307/248922

[54] Shrestha N. Factor analysis as a tool for survey analysis. American journal of applied mathematics and statistics. 2021 Jan 20;9(1):4–11. doi: 10.12691/ajams-9-1-2

[55] Rajapakshe W, Karunaratna DS, Ariyaratne WH, Madushika HL, Perera GS, Shamila P. Aggressive strategies of the COVID-19 pandemic on the apparel industry of Sri Lanka using structural equation modeling. PLoS ONE. 2023;18(6): doi: 10.1371/journal.pone.0286717 37343038 PMC10284376

[56] Marsh HW, Balla JR, McDonald RP. Goodness-of-fit indexes in confirmatory factor analysis: The effect of sample size. Psychological bulletin. 1988;103(3):391. https://psycnet.apa.org/record/1989-14212-001

[57] Browne M. W. Robert C. Alternative Ways of Assessing Model Fit. Sociological Methods & Research, (1992). doi: 10.1177/0049124192021002005

[58] Hu LT, Bentler PM. Cutoff criteria for fit indexes in covariance structure analysis: Conventional criteria versus new alternatives. Structural equation modeling: a multidisciplinary journal. 1999;6(1):1–55. doi: 10.1080/10705519909540118

[59] Bentler PM, Bonett DG. Significance tests and goodness of fit in the analysis of covariance structures. Psychological bulletin. 1980;88(3):588. https://psycnet.apa.org/record/1981-06898-001

[60] Burnes B. The origins of Lewin’s three-step model of change. The Journal of Applied Behavioral Science. 2020;56(1):32–59. doi: 10.1177/0021886319892

[61] Rafferty AE, Jimmieson NL, Armenakis AA. Change readiness: A multilevel review. Journal of management. 2013;39(1):110–35. doi: 10.1177/01492063124574

[62] Oreg S, Bartunek JM, Lee G, Do B. An affect-based model of recipients’ responses to organizational change events. Academy of Management Review. 2018;43(1):65–86. doi: 10.5465/amr.2014.0335

[63] Chappell S, Pescud M, Waterworth P, Shilton T, Roche D, Ledger M, et al. Exploring the process of implementing healthy workplace initiatives: mapping to Kotter’s leading change model. Journal of occupational and environmental medicine. 2016;58(10):e341–8. doi: 10.1097/JOM.0000000000000854 27525528

[64] Boies K, Fiset J, Gill H. Communication and trust are key: Unlocking the relationship between leadership and team performance and creativity. The leadership quarterly. 2015;26(6):1080–94. doi: 10.1016/j.leaqua.2015.07.007

[65] Saeed I, Khan J, Zada M, Zada S. Employee sensemaking in organizational change via knowledge management: leadership role as a moderator. Current Psychology. 2024;43(7):6657–71. doi: 10.1007/s12144-023-04849-x

[66] Men LR, Yue CA, Liu Y. Vision, passion, and care: The impact of charismatic executive leadership communication on employee trust and support for organizational change. Public Relations Review. 2020;46(3):101927. doi: 10.1016/j.pubrev.2020.101927

[67] Cameron E, Green M. Making sense of change management: A complete guide to the models, tools and techniques of organizational change. Kogan Page Publishers; 2019.

[68] Northouse PG. Leadership: Theory and practice. Sage publications; 2021.

[69] Adams BG, Meyers MC, Sekaja L. Positive leadership: Relationships with employee inclusion, discrimination, and well‐being. Applied Psychology. 2020 Sep;69(4):1145–73. doi: 10.1111/apps.12230

[70] Yammarino FJ, Cheong M, Kim J, Tsai CY. Is leadership more than “I like my boss”?. In Research in personnel and human resources management 2020, 38: 1–55. Emerald Publishing Limited.

[71] Lazzara EH, Benishek LE, Hughes AM, Zajac S, Spencer JM, Heyne KB, et al. Enhancing the organization’s workforce: Guidance for effective training sustainment. Consulting Psychology Journal: Practice and Research. 2021;73(1):1. https://psycnet.apa.org/buy/2021-30061-001

[72] Noe R, Hollenbeck J, Gerhart B, Wright P. Human Resources Management: Gaining a Competitive Advantage, Tenth Global Edition. New York, NY, USA: McGraw-Hill Education; 2006.

[73] Ratcliff CL, Wicke R, Harvill B. Communicating uncertainty to the public during the COVID-19 pandemic: A scoping review of literature. Annals of the International Communication Association. 2022;46(4):260–89. doi: 10.1080/23808985.2022.2085136

[74] Bansal A. Training during transitions: the context of a developing economy. In Advances in mergers and acquisitions 2017; 16: 115–131. Emerald Publishing Limited.

[75] Berkow K. Importance of effective leadership for the success of mergers and acquisitions. Pepperdine University; 2017. https://www.proquest.com/docview/1933004701?pq-origsite=gscholar&fromopenview=true&sourcetype=Dissertations%20&%20Theses

[76] Boykov D, Davitkovic M. Change in Organizations As A Tool For Organizational Development. Knowledge-International Journal. 2023 Dec 10;61(1):193–7. https://ikm.mk/ojs/index.php/kij/article/view/6402

[77] Asdar M. Strategies for Managing Employees during Mergers and Acquisitions. Advances in Human Resource Management Research. 2023;1(3):114–25. doi: 10.60079/ahrmr.v1i3.200

