# Correction: Enhancing Mergers and Acquisitions (M&A) Performance: Analyzing the Role of Human Resource Practices in Sri Lanka's Telecommunication Industry Through Lewin’s Change Management Model

**DOI:** 10.1371/journal.pone.0347909

**Published:** 2026-04-21

**Authors:** S. H. N. Pubodhya, Wasantha Rajapakshe

## Notice of Republication

This article was republished on March 13, 2026, to remove an error in the abstract. Please download this article again to view the correct version.
