# Supplementary figures and images for: Enhancing Mergers and Acquisitions (M&A) performance: Analyzing the role of human resource practices in Sri Lanka’s telecommunication industry through Lewin’s change management model
Source: PLoS One. 2025 Jan 10;20(1):e0317117. doi: 10.1371/journal.pone.0317117 (PMC11723548; doi:10.1371/journal.pone.0317117)

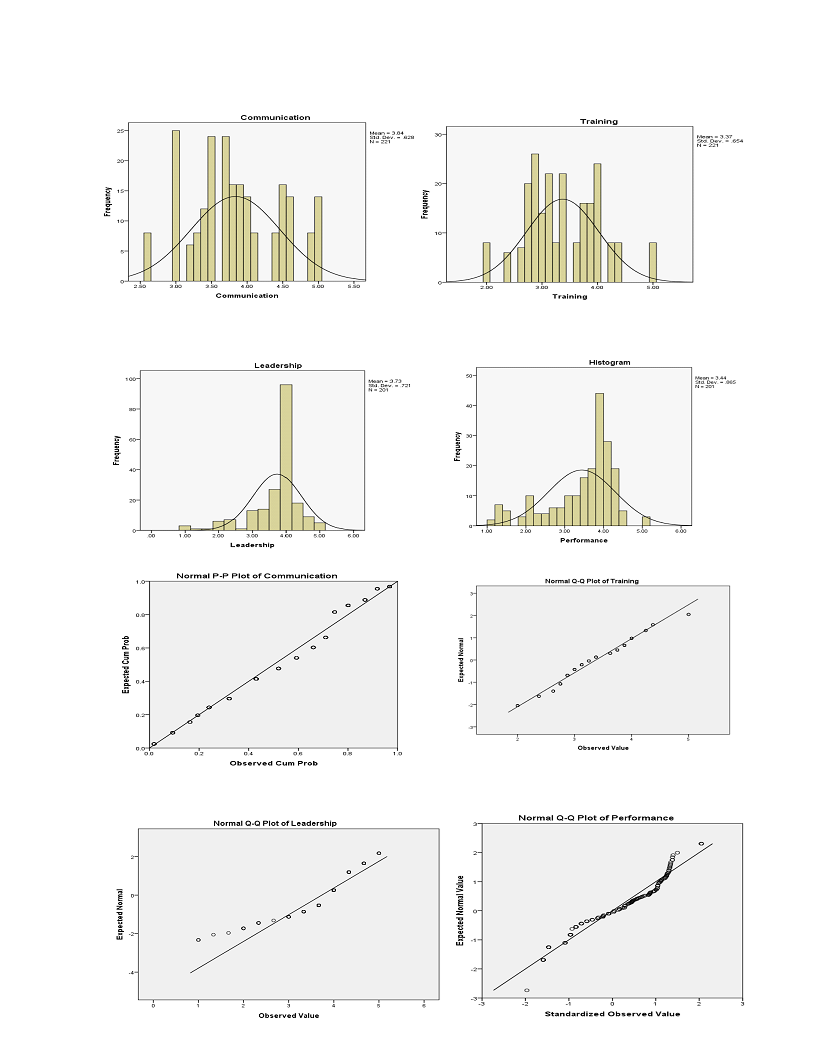

Supplement: S1 Fig — (TIF) [file pone.0317117.s001.tif]
